# Comparison of different microbiological procedures for the diagnosis of *Pneumocystis jirovecii* pneumonia on bronchoalveolar-lavage fluid

**DOI:** 10.1186/s12866-022-02559-1

**Published:** 2022-05-21

**Authors:** Iacopo Franconi, Alessandro Leonildi, Gianluca Erra, Roberta Fais, Marco Falcone, Emilia Ghelardi, Antonella Lupetti

**Affiliations:** 1 Department of Traslational Research and of New Technologies in Medicine and Surgery, University of Pisa, Via San Zeno 37, 56127 Pisa, Italy; 2 Department of Clinical and Experimental Medicine, University of Pisa, Pisa, Italy

**Keywords:** *Pneumocystis jirovecii*, Grocott-Gomori’s methenamine silver-staining, (1–3) β-D-glucan assay, Quantitative real-time PCR, End-point PCR, *Pneumocystis jirovecii* pneumonia, Bronchoalveolar-lavage fluid

## Abstract

**Background:**

The current diagnostic gold standard for *Pneumocystis jirovecii* is represented by microscopic visualization of the fungus from clinical respiratory samples, as bronchoalveolar-lavage fluid, defining “proven” *P. jirovecii* pneumonia, whereas qPCR allows defining “probable” diagnosis, as it is unable to discriminate infection from colonization. However, molecular methods, such as end-point PCR and qPCR, are faster, easier to perform and interpret, thus allowing the laboratory to give back the clinician useful microbiological data in a shorter time. The present study aims at comparing microscopy with molecular assays and beta-D-glucan diagnostic performance on bronchoalveolar-lavage fluids from patients with suspected *Pneumocystis jirovecii* pneumonia. Bronchoalveolar-lavage fluid from eighteen high-risk and four negative control subjects underwent Grocott-Gomori’s methenamine silver-staining, end-point PCR, RT-PCR, and beta-D-glucan assay.

**Results:**

All the microscopically positive bronchoalveolar-lavage samples (50%) also resulted positive by end-point and real time PCR and all, but two, resulted positive also by beta-D-glucan quantification. End-point PCR and RT-PCR detected 10 (55%) and 11 (61%) out of the 18 samples, respectively, thus showing an enhanced sensitivity in comparison to microscopy. All RT-PCR with a Ct < 27 were confirmed microscopically, whereas samples with a Ct ≥ 27 were not.

**Conclusions:**

Our work highlights the need of reshaping and redefining the role of molecular diagnostics in a peculiar clinical setting, like *P. jirovecii* infection, which is a rare but also severe and rapidly progressive clinical condition affecting immunocompromised hosts that would largely benefit from a faster diagnosis. Strictly selected patients, according to the inclusion criteria, resulting negative by molecular methods could be ruled out for *P. jirovecii* pneumonia.

## Introduction

*Pneumocystis jirovecii* is an atypical fungus causing Pneumocystis Pneumonia (PCP) in immunocompromised subjects, AIDS and/or immunosuppressive treatments [1, 2]. Clinical history and presentation along with radiological examinations drive presumptive diagnosis and empirical therapy. However, the role of laboratory tests has progressively grown in confirming it [3, 4].

The current diagnostic gold standard for *P. jirovecii* is represented by microscopic visualization of the fungus from clinical respiratory samples, as bronchoalveolar-lavage (BAL) fluid. Drawbacks of this technique include low sensitivity, results dependent on microbiologist’s experience, time-consuming [5, 6]. Due to its suboptimal sensitivity, negative microscopy cannot exclude infection.

Faster and more accurate detection of *P. jirovecii* can be provided by PCR-based assays, e.g. end-point and quantitative real-time PCR (qPCR) [6–9], which are more sensitive than microscopy, but cannot discriminate between colonization and PCP.

Serum (1–3) β-D-glucan (BDG) assay measures the level of a cell-wall component of many fungi, with the exceptions of Zygomycetes, *Blastomyces dermatitidis* and *Cryptococcus* spp., which may synthesize extremely low levels of BDG or not at all. Therefore, this assay lacks specificity, as BDG serum levels can rise due to various fungal infections, e.g. *Candida* spp. colonization and/or infection, but also for the presence of not fungi-related interfering factors, like intravenous treatment with antibiotics, albumin, immunoglobulin, and haemodialysis. Serum BDG might help the clinician to exclude the diagnosis of invasive fungal infections (IFD), like PCP, due to its high negative predictive value (93%) [10–14], although several authors have pointed out that this test cannot be considered the only test to perform in order to rule out the diagnosis of both IFD and/or PCP [15, 16]. BDG can be measured also in BAL, however, its role in clinical practice is still a matter of debate.

The aim of the present study was to compare diagnostic performance of end-point PCR and RT-PCR and BDG assay examinations with reference standard Grocott-Gomori’s methenamine silver-staining on BAL from patients with suspected PCP.

## Results

Based on the inclusion criteria, 18 patients with suspected PCP and four control patients were selected. One BAL sample/patient was analysed by the different methods (Table 1). Microscopy by GMS allowed to identify *P. jirovecii* in 9 (50%) of the 18 samples and molecular analysis by end-point Unyvero-HPN detected 10 (55.6%) positive samples. *P. jirovecii* was visualized microscopically in the same 9 samples categorized as intensely positive by end-point PCR. Differently, the sample n. 14, categorized as weakly positive by end-point PCR, was not visualized microscopically. BDG was measured in all samples. Fifteen (83.3%) out of 18 showed positive results, with 7 among those diagnosed by GMS and end-point PCR showing values from 591 to > 1000 pg/mL BDG.

**Table 1:** Comparison of microscopic, end-point PCR, RT-PCR, and β-D-glucan assays between PCP-patients and negative controls

Patients with suspected PCP	*P. jirovecii* detection by:			
	Methenamine-silver staining microscopy	Unyvero-HPN end-point PCR^a^	Sacace-P. jirovecii Real-TM PCR (Ct)^b^	β-D-glucan assay (pg/mL)^c^
1	+	+ + +	+ (20)	621
2	-	-	+ (28)	> 1000
3	+	+ + +	+ (21)	879
4	+	+ + +	+ (21)	960
5	+	+ + +	+ (22)	> 1000
6	-	-	-	> 1000
7	-	-	-	> 1000
8	-	-	-	> 1000
9	-	-	-	> 1000
10	+	+ + +	+ (26)	> 1000
11	+	+ + +	+ (24)	> 1000
12	-	-	-	> 1000
13	+	+ + +	+ (18)	591
14	-	+	+ (30)	118
15	-	-	-	> 1000
16	-	-	-	837
17	+	+ + +	+ (19)	255
18	+	+ + +	+ (19)	< 10
Total	9 (50%)	10 (55.6%)	11 (61.0%)	15 (83.3%)
Negative control patients				
19	-	-	-	309
20	-	-	-	< 10
21	-	-	-	115
22	-	-	-	< 10

^a^ + weakly, +  + moderately, +  +  + intensely positive samples

^b^(Ct) stands for threshold cycle value

^c^Results were < 400 pg/mL = negative; 400–450 pg/mL = indeterminate; > 450 pg/mL = positive

*P. jirovecii* DNA amplification by RT-PCR (Sacace *P. jirovecii* Real-TM®) detected 11 (61.0%) of the 18 samples. The same 9 samples already detected by GMS and end-point PCR showed a Ct value < 27, the additional sample detected only by end-point PCR (n. 14) resulted positive with Ct = 30, and a further sample (n. 2) with Ct = 28 was not detected by GMS nor end-point PCR. Interestingly, the BAL samples n. 17 and 18, which resulted positive microscopically and by end-point PCR, both with a Ct = 19 by RT-PCR, showed a negative BAL BDG result (255 pg/mL and < 10 pg/mL, respectively), which was performed in duplicate. For these patients, serum BDG, received on the same day, of patient n. 17 resulted negative (< 10 pg/mL) as well, whereas of patient n. 18 resulted positive (168 pg/mL). In sample n. 14, positive at molecular methods, the BAL BDG resulted negative (118 pg/mL). The sample n. 2 presented high BDG level (> 1000 pg/mL). Control patients resulted negative to each assay. A Kappa agreement analysis was performed showing a good strength of agreement (k = 0.780) between end-point PCR and the reference standard (GMS), a moderate strength of agreement (k = 0.576) between RT-PCR and the reference standard, and a poor strength of agreement (k =—0.33) between BDG on BAL and the reference standard. On secondary analysis a very good strength of agreement (k = 0.818) was found between the two PCR methods.

## Discussion

To the best of our knowledge, this is the first study comparing BDG assay with GMS microscopy and both end-point PCR and RT-PCR for *P. jirovecii* detection in BAL samples.

Direct microscopic staining is considered the gold standard for the diagnosis of “proven” PCP differently from qPCR, which allows to define “probable” PCP [17, 18]. However, molecular methods, such as end-point PCR and qPCR, require less man-hours, are faster, easier to perform and interpret, thus allowing the laboratory to give back the clinician useful microbiological data in a shorter time. In addition, the positive result by molecular methods can play an important role also for microscopic examination, as the latter can be difficult to interpret above all if the microbial load is low. The present study highlights that careful selection of patients with strict inclusion criteria is essential to define both an appropriate request and to save healthcare facility resources.

In this study, all the microscopically positive BAL samples (50%) also resulted positive by end-point and real time PCR and all, but two, resulted positive also by BDG quantification. As expected, RT-PCR was more sensitive than microscopy, allowing to detect *P. jirovecii* in 61% of the tested samples. In agreement with previous studies, positive results by RT-PCR with Ct < 27 were confirmed microscopically [19].

Levels of BDG were found to be high (≥ 837.3 pg/mL) in 6 patients with no evidence of *P. jirovecii* infection. Since BDG is present in the cell-wall of many fungi, BDG cannot be used to diagnose a specific fungal infection, thus indicating that BAL BDG should only be taken into consideration for its high negative predictive value [13]. Indeed, it resulted useful in ruling out the diagnosis of PCP for all the negative control patients. Moreover, this test is considered even more reliable in HIV positive patients to rule out PCP. However, our data indicate that negative BAL BDG as well as negative serum BDG values cannot exclude “proven” PCP infection in accordance with current literature [15, 16]. Indeed, patient n. 17 who had both negative BAL and serum BDG and was HIV positive, and patient n. 18 who had negative BAL and positive serum BDG and was HIV positive confirm that BDG test alone is not sufficient to rule out PCP even among high-risk patients as HIV positive patients [16].

Together, diagnosis of PCP can be upgraded if an appropriate microbiological test result becomes positive. According to the guidelines [17, 18], appropriate host factors, clinical and radiologic criteria should be confirmed by microscopy and qPCR. Although the present data are too limited to draw firm conclusions, we propose that patients strictly selected according to the inclusion criteria and resulting negative by molecular methods could be ruled out for PCP diagnosis. This conclusion is based on the following findings. First, the positive results obtained by GMS were confirmed by molecular methods, e.g., end-point PCR and RT-PCR. Second, RT-PCR showed an enhanced sensitivity in comparison to GMS and end-point PCR. Noteworthy, in comparison to GMS, molecular methods require reduced man-hour and turnaround time, and provide results independent from microbiologist’s experience. Third, in agreement with Fauchier et al. [19], all patients resulted positive by RT-PCR showing a Ct < 27 were confirmed microscopically, thus suggesting that these patients could be considered affected from PCP. Further studies are needed to determine PCR cut-off values to discriminate “proven” from “probable” PCP and from colonization, which would allow providing clinicians faster and reliable results.

## Conclusions

In conclusion, our work highlights the need of reshaping and redefining the role of molecular diagnostics in a peculiar clinical setting, like *P. jirovecii* infection, which is a rare but also severe and rapidly progressive clinical condition affecting immunocompromised hosts that would largely benefit from a faster diagnosis.

## Methods

This is a prospective methodological study. This study was conducted at the Pisa University Hospital, Mycology Unit, from January 2020 to October 2020.

### Inclusion criteria

Current immunosuppression (AIDS or immunosuppressive treatments), new-onset progressive exertional dyspnoea, fever, cough and hypoxia, a suggestive imaging with ground glass opacities, diffuse infiltrates and/or nodules, high lactate dehydrogenase levels, and no response to empirical antibiotic treatment [3, 4, 17, 18].

Four patients who could not meet the overmentioned criteria were used as negative controls.

### Microbiological diagnosis of *P. jirovecii*

Reference standard was Grocott-Gomori’s methenamine silver-staining. BAL samples were centrifuged (4000 × rpm, 10 min) and divided into two aliquots, one immediately used for microscopy and end-point PCR, one frozen (-20 °C) for further RT-PCR and BDG quantification.

All samples underwent: i) microscopic examination via Grocott-Gomori’s methenamine silver-staining (GMS); ii) end-point PCR Curetis Unyvero®–HPN (Hozgerlingen, Germany), target amplified gene was the 26S rDNA. BAL fluid (180 µL) was lysed in the Unyvero Sample Tube, and together with the Master Mix set within the cartridge inside the Unyvero Analyzer. Positivity to DNA search was expressed as weakly, moderately, intensely positive samples. End-point PCR analytical sensitivity regarding *P. jirovecii* detection was 10^5^ pathogens/mL, diagnostic sensitivity and specificity reported by manufactures’ instructions specifically for *P. jirovecii* were both 100%. This assay is performed with a commercially available kit that has overcome quality controls as required by CE approval; iii) *P. jirovecii* DNA extraction was performed by QIAamp DNA minikit (Qiagen GmbH, Hilden, Germany) and amplification by RT-PCR (Sacace Pneumocystis jirovecii Real-TM®, Sacace Biotechnologies, Como, Italy) with fluorescent reporter dye probes specific for *P. jirovecii* and internal control (β-globin gene) used as an amplification control for each specimen and to identify possible reaction inhibition. The target amplified gene was the 26S rDNA. Positive/negative controls were tested along with the patient’s sample, following manufacturer's instructions in a 96-well plate on CFX96 Touch Real-Time PCR Detection System (Bio-Rad Laboratories, Hercules CA, USA). Samples were considered positive if the threshold cycle (Ct) value was ≤ 38 and curves showed the typical sigmoidal profile. RT-PCR (Sacace Pneumocystis jirovecii Real-TM®) analytical sensitivity was 200 DNA copies/mL, both sensitivity and specificity were 100%. The method used is qualitative, nevertheless, through Ct values, Real-Time PCR allows an estimation of the fungal burden in study samples as previously described [18, 20]. This assay is performed with a commercially available kit that has overcome quality controls as required by CE approval; iv) thawed BAL samples were centrifuged (3000 × rpm, 10 min) and supernatants used for BDG assay via Category Goldstream®– Product name: Fungus (1–3) β-D-Glucan Test Chromogenic Method (GKT-5 M) (Era Biology, Tianjin, China). BDG was quantified by a kinetic automatic reader (IGL-200, Era Biology, Tianjin, China), and compared with standard curves. Results were < 400 pg/mL = negative; 400–450 pg/mL = indeterminate; > 450 pg/mL = positive. Regarding *P. jirovecii* infection serum BDG assay showed a pooled sensitivity of 87% (95% CI: 0.73–0.94), a specificity of 97% (95% CI: 0.87–0.99), a positive predictive value of 97% (95% CI: 0.85–0.99), and a negative predictive value of 88% (95% CI: 0.75–0.95) [9]. This assay is performed with a commercially available kit that has overcome quality controls as required by CE approval.

## Data Availability

All data are presented in the manuscript.
